# Cyclophotocoagulation in Neovascular Glaucoma with Near-Total Synechial Angle Closure

**DOI:** 10.1155/2023/5719002

**Published:** 2023-10-26

**Authors:** Jessie Wang, Lindsay Y. Chun, Mary Qiu

**Affiliations:** Department of Ophthalmology & Visual Science, University of Chicago, Chicago, IL, USA

## Abstract

**Objective:**

To describe a single surgeon's experience utilizing prompt primary slow-burn transscleral cyclophotocoagulation (CPC) with prior or concurrent anti-VEGF and subsequent aqueous shunt as needed in NVG eyes with near-total synechial angle closure at presentation.

**Methods:**

Retrospective chart review of all NVG patients with uncontrolled IOP, active anterior segment NV, near-total synechial angle closure, and no contraindications to prompt anti-VEGF who received CPC within 3 days of presentation with at least 6 months of follow-up.

**Results:**

Eight patients with mean age 60.6 years were included. Underlying etiologies were CRVO (*N* = 3), PDR (*N* = 2), CRAO (*N* = 1), BRVO (*N* = 1), and chronic RD (*N* = 1). All eyes underwent CPC with intravitreal anti-VEGF within 3 days of presentation. Five patients did not require subsequent aqueous shunts through a mean follow-up of 15 months; most recent visual acuities ranged from 20/40 to LP, and IOPs ranged from 5 to 11 mmHg on 0 to 3 IOP-lowering medications. Three patients who required subsequent tubes had complete regression of active anterior segment NV at the time of surgery. Most recent visual acuities ranged from 20/100 to 20/125, and IOPs ranged from 8 to 14 mmHg on 0 meds at a mean follow-up of 10 months. No eyes developed uncontrolled inflammation, sympathetic ophthalmia, or phthisis.

**Conclusion:**

Prompt primary slow-burn CPC with prior or concurrent anti-VEGF may be an effective strategy to immediately lower IOP in acute NVG eyes with active anterior segment NV and near-total synechial angle closure. If IOP becomes uncontrolled later, an aqueous shunt can be implanted in a controlled setting after active anterior segment NV has regressed.

## 1. Introduction

Neovascular glaucoma (NVG) is an aggressive secondary glaucoma caused by retinal ischemia. Its pathophysiology is mediated by vascular endothelial growth factors (VEGF), which exerts an angiogenic effect, leading to fibrovascular membrane growth over the iridocorneal angle and subsequent aqueous outflow obstruction. Early on, intraocular pressure- (IOP-) lowering medications can still be effective, even with active angle neovascularization (NVA) [1, 2]. As the disease progresses, myofibroblasts cause contraction and progressive synechial angle closure, rendering IOP-lowering medications ineffective [1–3].

Achieving safe and timely IOP control in the setting of acute NVG with active NVA and synechial angle closure poses several challenges. While prompt intravitreal anti-VEGF rapidly regresses NV, this does not translate to an appreciable IOP reduction. Wakabayashi et al. reported that NVG eyes respond differently to anti-VEGF depending on the angle status. In eyes with <75% PAS, intravitreal anti-VEGF led to rapid regression of anterior segment neovascularization with normalization of IOP in 71% of eyes. However, in eyes with near-total angle closure (≥75% PAS), though intravitreal anti-VEGF led to regression of neovascularization, the IOP remained elevated; 93% of these eyes required emergent IOP-lowering procedures [2].

Classically, aqueous shunts have been favored in eyes with good visual potential, and cyclophotocoagulation (CPC) has been reserved for eyes with poor visual potential, due to concerns of CPC causing uncontrolled inflammation and phthisis [4]. However, implanting aqueous shunts into eyes with active NV carries an increased risk of bleeding-associated complications and poorer long-term success [5]. Concurrent cataract surgery may also optimize visual outcomes and facilitate sulcus tube placement, which carries a lower risk of corneal endothelial cell loss as compared to anterior chamber tube placement [6], but may also be too high-risk in eyes with NV.

The recently introduced, gentler “slow-burn” CPC setting demonstrates equivalent efficacy with lower rates of prolonged inflammation, compared to traditional CPC settings. “Slow-burn” CPC utilizes lower energy settings at longer durations to achieve a gentler and less inflammatory, but still equivalently efficacious, effect [7]. As a nonincisional option, CPC provides immediate IOP-lowering in cases where medical therapy is ineffective and incisional surgery risks bleeding-associated complications, while allowing time for adjunctive antineovascular treatments to take effect. While prior studies describe primary CPC for NVG, they do not stratify by angle status. This is the first report to propose customizing acute NVG treatment based on angle status, to focus solely on NVG eyes with near-total synechial angle closure, and to advocate for “slow-burn” CPC in eyes of all visual potential.

## 2. Case Presentation

### 2.1. Materials and Methods

Retrospective chart review was performed for all eyes with NVG that underwent “slow-burn” CPC with a single surgeon (MQ) between 10/1/2019–4/1/2022. Eyes were included if the angle had near-total synechial closure (≥75% PAS) at the time of presentation, CPC and intravitreal anti-VEGF occurred within 3 days of presentation, and there were ≥6 months of follow-up. A cutoff of ≥75% PAS was used to define near-total synechial angle closure, as this cutoff has been previously used in literature to differentiate between “closed-angle” and “open-angle” NVG [2]. Exclusion criteria included contraindications to intravitreal anti-VEGF or anterior chamber paracentesis, prior aqueous shunt, trabeculectomy, minimally invasive subconjunctival/suprachoroidal device, or cyclodestructive procedure.

All transscleral CPCs were performed with the Iridex-Cyclo-G6-Glaucoma-Laser-System and G-probe (IRIDEX Corporation, Mountain View, California) with a retrobulbar block (5 patients) or general anesthesia (3 patients—one monocular and two on aspirin). All patients underwent “slow burn” settings with energy ranging from 1100 to 1500 mW (starting at 1250 mW), duration 4 seconds per spot, and 16-24 spots per surgeon discretion. After CPC, a peribulbar injection of dexamethasone 10 mg was administered in the inferior fornix. Postoperatively, patients used prednisolone every 2 hours while awake and atropine twice daily, tapered over at least 10 weeks. CPC was not repeated for any patients, as the objective was to lower the IOP in the acute phase to allow for neovascularization to regress, so that if needed, an aqueous shunt can be implanted in a quiet, controlled eye.

### 2.2. Results

Eight patients were included in this analysis; baseline demographics, clinical characteristics, and clinical course after CPC are shown in Table 1. To date, 5/8 (63%) patients have maintained adequate IOP control after initial CPC without the need for subsequent aqueous shunt through a mean follow-up of 17 months (range 6-27 months); each patient's most recent VA, IOP, and number of medications are shown in Table 1. Notably, one eye attained 20/40 vision after CPC (from 20/300 preoperatively), suggesting that “slow burn” CPC has utility in eyes with good visual potential, and that moreover, assessing visual potential in the acute setting can be challenging. The other 3/8 (37%) patients required subsequent aqueous shunts (all Baerveldt-350 with 3-0 Prolene ripcord). The initial CPC provided transient IOP-lowering while anti-VEGFs and PRP exerted their antineovascular effects, so that the eventual tubes could be inserted in a more controlled setting. Incisional surgery occurred between POW11-41, and two patients underwent concurrent cataract surgery to facilitate sulcus tube placement. At most recent follow-up, VA ranged from 20/100 to 20/125, and IOP ranged from 8 to 14 mmHg on zero medications. All eyes attained stable or improved VA, and none developed new-onset macular edema, uncontrolled inflammation, sympathetic ophthalmia, or phthisis. Absence of macular edema was verified with macular optical coherence testing (OCT).

## 3. Discussion

Urgent IOP-lowering surgery is usually needed in NVG eyes with near-total synechial angle closure, despite successful NV regression [1–3]. Most patients in this case series achieved adequate IOP control with “slow-burn” CPC alone, though longer follow-up is needed to ascertain whether they will eventually require aqueous shunts. More recent studies have demonstrated the improved safety profile of “slow-burn” settings, allaying the historical safety concerns associated with traditional CPC [7, 8]. For the three eyes requiring subsequent aqueous shunts, no intraoperative bleeding-related complications occurred. Per the authors' treatment protocol, primary “slow-burn” CPC is not intended to avoid a tube indefinitely but is instead intended to postpone it until NV regression and to facilitate sulcus tube placement. Thus, if a tube is needed later, neither patients nor physicians consider it to be a failure of CPC, but rather the next step of an optimized multistep treatment course.

There is growing evidence supporting that NVG treatments and outcomes may differ depending on angle status, such as the aforementioned study by Wakabayashi et al. [2]. Another recent case report demonstrated that NVG eyes with entirely open angles may achieve normalized IOP with antineovascular therapy alone [9], and NVG eyes with partially open angles (<75% PAS) may achieve normalized IOP with a combination of antineovascular therapy and microinvasive gonioscopy-assisted transluminal trabeculotomy (GATT), which restores physiologic aqueous outflow through the conventional outflow pathway [10]. In contrast, eyes in this case series presented with near-total (≥75% PAS) synechial angle closure. As expected, antineovascular therapy was ineffective at controlling the IOP despite NV regression, and GATT could not be performed since the trabecular meshwork was inaccessible due to synechial angle closure. Therefore, an urgent nonincisional IOP-lowering therapy was needed to avoid inserting tubes into eyes with active neovascularization.

Limitations to this study include its retrospective nature, which hold the potential for bias, confounding variables, inability to control for exposure, and limited data availability. Future larger-scale studies are needed to further investigate the optimal treatment protocol for NVG eyes presenting with near-total synechial angle closure.

The authors propose a novel algorithm to evaluating and treating NVG eyes by selecting an IOP-lowering intervention based on angle anatomy, as prior research has demonstrated that NVG eyes respond differently to anti-VEGF depending on the angle status, with synechially closed eyes being less responsive to medical IOP-lowering and necessitating more surgical interventions [2]. In cases of corneal edema barring adequate gonioscopic examination, eyes responding to medical therapy with corneal clearing may be presumed to have an at least partially open angle, whereas eyes with persistent corneal edema and elevated IOP unresponsive to medical therapy may be presumed to have near-total angle closure. This study advocates that for NVG eyes with active anterior segment neovascularization and near-total synechial angle closure, prompt primary CPC with “slow-burn” settings and judicious peri- and postoperative steroids, adjunctive anti-VEGFs and PRP, and a plan for a subsequent aqueous shunt, if needed, is an effective treatment strategy. Collaborative communication between the patient, glaucoma specialist, and retina specialist and close long-term follow-up are critical to implementing a multidisciplinary treatment plan and optimizing outcomes.

## Figures and Tables

**Table 1:** Demographics, clinical characteristics, and postoperative outcomes in NVG eyes with near-total synechial angle closure and active anterior segment neovascularization treated with prompt primary “slow-burn” cyclophotocoagulation and subsequent aqueous shunt as-needed.

Age (yrs)	Etiology	Presenting VA	Presenting IOP (mmHg)	Time btw presentation and anti-VEGF #1 (days)	Time btw presentation and CPC (days)	IOP and # of medications within 1 week of CPC	# of anti-VEGF injections since presentation	# of PRP lasers since presentation	Time btw CPC and tube (wks)	IOP and # of medications prompting tube	Concurrent cataract surgery	BCVA at last follow-up	IOP, # of medications, and follow-up duration
60s	Chronic RD	HM	36	0	3	23 mmHg on 4 meds	1	0	—	—	—	HM	6 mmHg on 0 meds at 18 months
60s	PDR	20/300	60	0	2	5 mmHg on 0 meds	4	2	—	—	—	20/40	11 mmHg on 0 meds at 15 months
70s	RVO	LP	55	0	0	6 mmHg on 1 med	7	0	—	—	—	LP	5 mmHg on 1 med at 27 months
60s	RVO	HM	50	2	2	7 mmHg on 4 meds	5	3	—	—	—	CF	9 mmHg on 3 meds at 13 months
70s	RAO	LP	31	2	2	5 mmHg on 2 meds	2	3	—	—	—	HM	5 mmHg on 0 med at 11 months
50s	RVO	HM	68	0	0	10 mmHg on 4 meds	5	4	41	25 mmHg on 4 meds	Yes	20/100	8 mmHg on 0 meds at 15 months
50s	RVO	20/300	49	0	2	20 mmHg on 0 meds	6	1	11	65 mmHg on 3 meds	No	20/125	8 mmHg on 0 meds at 9 months
20s	PDR	20/1250	68	0	0	27 mmHg on 0 meds	6	2	11	26 mmHg on 5 meds	Yes	20/125	14 mmHg on 0 meds at 6 months

All patients presented initially with IOPs on zero IOP-lowering medications. All aqueous shunts were Baerveldt-350 with a 3-0 Prolene ripcord left in place indefinitely to prevent hypotony. IOP = intraocular pressure; VA = visual acuity; CPC = cyclophotocoagulation; anti-VEGF = antivascular endothelial growth factor; PRP = panretinal photocoagulation; BCVA = best corrected visual acuity; medications = IOP-lowering medications; RD = retinal detachment; PDR = proliferative diabetic retinopathy; RVO = retinal vein occlusion; RAO = retinal artery occlusion; HM = hand motion; LP = light perception.

## Data Availability

The retrospective data used to support the findings of this study are available from the corresponding author upon request.

## Additional Points

*Meeting Presentation*. ARVO 2022, Denver, CO; EGS 2022, Athens, Greece; WIO 2022, Monterey, CA; APGC 2022, Kuala Lumpur, Malaysia; WOC 2022.
